# Brain tissue oxygenation guided therapy and outcome in non-traumatic subarachnoid hemorrhage

**DOI:** 10.1038/s41598-021-95602-6

**Published:** 2021-08-10

**Authors:** Elisa Gouvea Bogossian, Daniela Diaferia, Narcisse Ndieugnou Djangang, Marco Menozzi, Jean-Louis Vincent, Marta Talamonti, Olivier Dewitte, Lorenzo Peluso, Sami Barrit, Mejdeddine Al Barajraji, Joachim Andre, Sophie Schuind, Jacques Creteur, Fabio Silvio Taccone

**Affiliations:** 1 Department of Intensive Care, Erasme Hospital, Université Libre de Bruxelles, Route de Lennik, 808, 1070 Brussels, Belgium; 2 Department of Neurosurgery, Erasme Hospital, Université Libre de Bruxelles, Route de Lennik, 808, 1070 Brussels, Belgium; 3 Department of Radiology, Erasme Hospital, Université Libre de Bruxelles, Route de Lennik, 808, 1070 Brussels, Belgium

**Keywords:** Neuro-vascular interactions, Biomarkers, Diseases, Medical research, Neurology

## Abstract

Brain hypoxia can occur after non-traumatic subarachnoid hemorrhage (SAH), even when levels of intracranial pressure (ICP) remain normal. Brain tissue oxygenation (PbtO_2_) can be measured as a part of a neurological multimodal neuromonitoring. Low PbtO_2_ has been associated with poor neurologic recovery. There is scarce data on the impact of PbtO_2_ guided-therapy on patients’ outcome. This single-center cohort study (June 2014–March 2020) included all patients admitted to the ICU after SAH who required multimodal monitoring. Patients with imminent brain death were excluded. Our primary goal was to assess the impact of PbtO_2_-guided therapy on neurological outcome. Secondary outcome included the association of brain hypoxia with outcome. Of the 163 patients that underwent ICP monitoring, 62 were monitored with PbtO_2_ and 54 (87%) had at least one episode of brain hypoxia. In patients that required treatment based on neuromonitoring strategies, PbtO_2_-guided therapy (OR 0.33 [CI 95% 0.12–0.89]) compared to ICP-guided therapy had a protective effect on neurological outcome at 6 months. In this cohort of SAH patients, PbtO_2_-guided therapy might be associated with improved long-term neurological outcome, only when compared to ICP-guided therapy.

Spontaneous SAH (SAH) is a life-threatening disease that can cause severe disabilities in the survivors^1–3^. Immediately after aneurysm rupture, an acute increase in intracranial pressure (ICP), together with a decrease in cerebral perfusion, can lead to brain ischemia^4^. This phenomenon is associated with endothelial damage, excitotoxicity and neuroinflammation, all resulting in neuronal death^5,6^. These processes identified as “early brain injury” (EBI), can contribute to the further increase in ICP that, if uncontrolled, will lead to severe cerebral injury and brain death^6–8^.

As such, ICP monitoring has been recommended, with the aim to early detect ICP elevation and potentially reduce early mortality^3,9,10^. However, tissue hypoxia can occur even when ICP remains within normal values^11–13^, so that ICP monitoring alone may not be sufficient to minimize cerebral ischemia in these patients. Adding brain tissue oxygenation (PbtO_2_) monitoring in a multimodal approach (MMM) to detect cerebral hypoxia and initiate early neuroprotective intervention may improve patients’ outcome^14–16^. Moreover, in a later phase of SAH, up to 30% of patients can develop delayed cerebral ischemia (DCI)^17^, which is an important determinant cause, together with EBI, of poor outcome^18–20^. Early recognition of DCI is essential for timely interventions to minimize brain damage. As clinical examination is often unreliable in these patients (persistent poor clinical condition since admission or use of sedative drugs associated with limited clinical manifestations), PbtO_2_ monitoring could help detect and treat brain hypoxia due to DCI^21,22^.

PbtO_2_ monitoring has been extensively studied in traumatic brain injury (TBI) patients, where brain hypoxia is associated with poor neurologic outcome and high mortality rates^13,23–25^. Moreover, some studies using PbtO_2_ guided-therapy have shown an improved neurological outcome when compared to ICP-guided therapy^26–28^. In SAH patients, low PbtO_2_ values have also been associated with adverse neurologic events, such as metabolic distress, cerebral vasospasm and DCI, as well as with poor neurologic outcome^22,29,30^. However, whether PbtO_2_-guided therapy improve patients’ outcome after SAH is still a matter of debate.

To assess this issue, the aim of this study was to investigate the impact of PbtO_2_ guided-therapy on the outcome of SAH patients. Our hypothesis was that PbtO_2_ guided-therapy would allow improved neurological outcome via an early diagnosis and treatment of secondary brain injuries.

## Methods

### Study design

We reviewed our cohort of patients with non-traumatic SAH treated from June 2014 until March 2020 in our Department of Intensive Care. This study was approved by the Erasme Hospital (Université Libre de Bruxelles) ethics committee (P2019/649) on May 23rd 2019, that waived the need for informed consent. All methods were carried out in accordance with relevant scientific and ethical guidelines and regulations.

All adult (> 18 years) patients admitted with non-traumatic SAH were eligible, provided that they needed an ICP monitoring within the first 48 h after admission. The sole exclusion criterion was imminent death, without any specific therapies and leading to early limitation of life-sustaining therapies. ICP monitoring was inserted in patients with an initial GCS < 9 or with clinical deterioration and hydrocephalus on cerebral CT-scan. All patients undergoing ICP monitoring were also eligible for PbtO_2_ monitoring; however, the decision to add a PbtO_2_ monitoring was dependent by the availability of the monitoring device (i.e. one device in 2014, only patients with GCS < 9 despite hydrocephalus treatment were therefore monitored; three devices since November 2017). Moreover, patients with delayed deterioration were also monitored with PbtO_2_ if they became unconscious and unable to obey commands (GCS < 9) or if they required sedation.

### Patient management and definitions

A detailed account of the management of SAH patients in our department can be found at supplementary text 1. Both ICP and PbtO_2_ (Integra Licox Brain Tissue Oxygen Monitoring System, Integra LifeSciences Services, Saint Priest, France) were measured in real-time and collected prospectively. Intracranial hypertension was defined by the observation of at least one ICP value above 20 mmHg for at least 5 min at any time. Brain tissue hypoxia was defined by a PbtO_2_ below 20 mmHg, and severe brain hypoxia by a value less than 10 mmHg^22^. We defined the “burden of hypoxia” as the area under the curve (PbtO_2_ × time, expressed as mmHg*hour) below 20 and 10 mmHg of PbtO_2_, respectively. In these SAH patients requiring invasive monitoring, the initial management was independent from ICP and PbtO_2_ values and included head position, avoidance of neck compression and extra-cerebral cerebral injuries (Supplemental Fig. 1A, B). ICP-guided therapy was considered as all specific therapeutic interventions (i.e. increased sedation, osmotic therapy, hyperventilation, high-dose barbiturates, decompressive craniectomy) aiming to achieve an ICP < 20 mmHg. PbtO_2_-guided therapy was considered as all specific therapeutic interventions (i.e. induced hypertension, changes in PaCO_2_, red blood cells transfusions, cerebral arteriography with chemical angioplasty) aiming to achieve a PbtO_2_ > 20 mmHg (Supplemental Fig. 1A, B).

### Data collection

We recorded demographic data, such as age, gender and presence of comorbidities. Clinical severity scores on admission, such as the Sequential Organ Failure Assessment (SOFA)^31^ and the Acute Physiology and Chronic Health Evaluation (APACHE) II scores, were computed^32^. Neurologic assessment scales and imaging scale on admission, such as the World Federation of Neurological Surgeons (WFNS) scale^33^, the Glasgow Coma Scale (GCS)^34^ and the modified Fisher grading scale^35^, were reported for all patients. Patients with WFNS 4 or 5 on admission were defined as “poor grade”; patients with modified Fisher scale 3 or 4 on admission were defined as “high risk” for cerebral vasospasm. We also recorded the type of intervention to secure the aneurysm (i.e. endovascular vs. surgical treatment), the various interventions that the patients received during the ICU stay (i.e. mechanical ventilation, vasopressor and inotropic support and renal replacement therapy) and the development of complications, including seizures, re-bleeding, cerebral vasospasm and DCI. We also recorded the specific treatments used to treat intracranial hypertension and/or tissue hypoxia. We recorded hospital mortality, the Glasgow Outcome Scale (GOS)^36^ at 6 months and the occurrence of unfavorable neurological outcome (UO), as defined by a GOS at 6 months of 1–3, using medical reports from follow-up visits.

### Study outcomes

We assessed the impact of ICP/PbtO_2_-guided therapy on neurological outcome in SAH patients. In particular, a subgroup analysis including only patients receiving therapies driven by neuromonitoring (ICP-guided vs. ICP/PbtO_2_-guided) was performed. Secondary outcomes included: (b) the impact of ICP/PbtO_2_ guided therapy on hospital mortality; (c) subgroup analysis of aneurysmal SAH patients.

### Statistical analysis

Descriptive statistics were computed for all variables. Numeric variables were described either as median and interquartile intervals 25–75% or mean and standard deviation. Categorical variables were described as proportions. We assessed the distribution pattern of each variable using the Kolmogorov–Smirnov test. Normally distributed continuous variables were compared using t Student test and asymmetrically distributed variables were compared using Mann–Whitney test. Categorical variables were analyzed using chi square or Fisher’s exact test, as appropriate. We performed a binary logistic regression to assess the association of ICP/PbtO_2_-guided therapy with UO, adjusted by clinically and statistically (*p* < 0.01 in the univariable analysis) relevant confounders. Similarly, we conducted a Cox regression to evaluate the association of ICP/PbtO_2_-guided therapy and hospital mortality, adjusted for confounders. In the subgroup of patients that received interventions based on MMM, we performed a logistic regression to assess a possible association between ICP/PbtO2 guided therapy compared to ICP guided therapy and neurological outcome in 6 months. Adjusted odds ratios (ORs) with 95% confidence intervals (CIs) were computed for all variables in all multivariable models. The independence of errors, presence of multicollinearity and the presence of influential outlier assumptions were checked and none of them were violated. As a sensitivity analysis, a similar statistical approach (adjusted Cox regression to evaluate the association of ICP/PbtO_2_ -guided therapy and hospital mortality; logistic regression analysis to assess the association between ICP/PbtO_2_ guided therapy compared to ICP guided therapy and neurological outcome in 6 months) was used to analyze only patients with aneurysmal SAH. All statistical analysis was done using the program SPSS 27.0 for MacIntosh. A *p* value < 0.05 was considered significant.

### Ethics approval and consent to participate

The study protocol was approved by the Erasme Hospital (Université Libre de Bruxelles) ethics committee (P2019/649) and the informed written consent was waived due to the retrospective design of the study. All methods were carried out in accordance with relevant scientific and ethical guidelines and regulations.

## Results

### Study population

**Figure 1:**
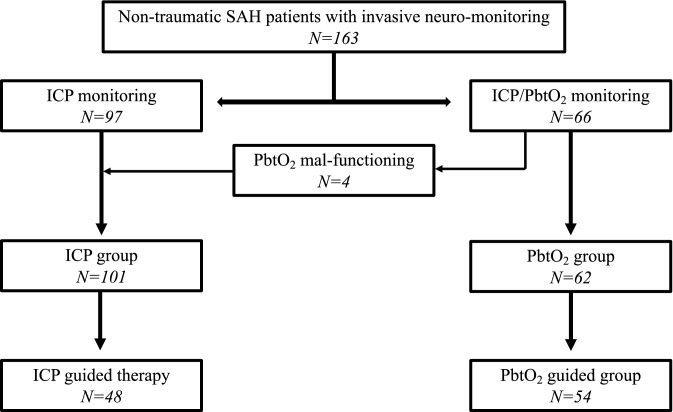
Flow-chart of the study. SAH: subarachnoid hemorrhage; ICP: intracranial pressure; PbtO_2_: brain tissue oxygenation.

Of a total of 322 patients admitted for non-traumatic spontaneous SAH, 168 were monitored with ICP monitoring. Five patients died within 48 h, so that 163 patients were included in the analysis: 97 monitored with ICP only and 66 with ICP and PbtO_2_ (ICP/PbtO_2_ group). However, 4 patients had malfunctioning/misplaced PbtO_2_ catheters and were eventually analyzed into the ICP group (Fig. 1). In the ICP only monitored group, 43/101 (43%) patients received ICP-guided therapy; in the ICP/PbtO_2_ group, 54/62 (87%) received ICP/PbtO_2_-guided therapy, of which 22 received treatment triggered only by PbtO_2_. Also, in the ICP/PbtO_2_ group, 5/62 (8%) patients received ICP but not PbtO_2_-guided therapy.

Patients were predominantly female (97/163, 60%) and had a mean age of 55 (± 13) years (Table 1); the median GCS on admission was 6 (3–12). A ruptured aneurysm was identified in 143/163 (88%) patients; in 8 (5%) patients SAH was peri-mesencephalic and the remaining 12 (7%) patients had no evident cause of bleeding (*sine materia*). Among the 143 aneurysmal SAH patients, 105 (74%) had an aneurysm located in the anterior circulation. The most common comorbidity was arterial hypertension (80/163, 49%) and hydrocephalus was the most common neurological complication (111/163, 68%). DCI occurred in 59 (36%) patients and intracranial hypertension in 95 (58%) patients during ICU stay. Sixty-eight patients (42%) died at hospital discharge and 111 (68%) patients had UO at 6 months.

**Table 1:** Characteristics of the studied population, according to the type of neuro-monitoring.

	All patients (N = 163)	ICP (N = 101)	ICP/PbtO_2_ (N = 62)	*p* value
**On admission**				
Age, mean (± SD)	55 (± 13)	55 (± 13)	54 (± 12)	0.42
Male gender, n (%)	66 (41)	41 (41)	25 (40)	0.99
APACHE score, median (IQR)	18 (13–21)	18 (13–21)	19 (13–22)	0.80
SOFA score, median (IQR)	7 (4–10)	7 (4–9)	8 (4–10)	0.42
GCS, median (IQR)	6 (3–12)	6 (3–11)	4 (3–13)	0.51
WFNS 4–5, n (%)	126 (77)	80 (79)	46 (74)	0.56
mFisher scale 3 or 4 points, n (%)	154 (95)	99 (98)	55 (89)	0.03
Intraparenchymal hematoma, n (%)	60 (37)	30 (30)	30 (48)	0.02
Ruptured aneurysm, n (%)	143 (88)	82 (81)	61 (98)	0.001
Anterior circulation aneurysm, n (%)	105 (64)	77 (76)	28 (45)	0.001
**Comorbidities**				
HAS, n (%)	80 (49)	55 (55)	25 (40)	0.11
DM, n (%)	18 (11)	13 (13)	5 (8)	0.44
Heart disease, n (%)	18 (11)	13 (13)	5 (8)	0.44
Previous neuro disease, n (%)	17 (10)	14 (14)	3 (5)	0.11
CKD, n (%)	4 (3)	3 (3)	1 (2)	0.99
Asthma/COPD, n (%)	17 (10)	11 (11)	6 (10)	0.99
Immunosuppression, n (%)	9 (6)	7 (7)	2 (3)	0.49
Cancer, n (%)	11 (7)	9 (9)	2 (3)	0.21
Cirrhosis, n (%)	7 (4)	5 (5)	2 (3)	0.71
**Support therapies during ICU stay**				
Vasopressor, n (%)	124 (76)	66 (65)	58 (94)	0.001
Inotropic, n (%)	45 (28)	9 (9)	36 (58)	0.001
Mechanical ventilation, n (%)	146 (90)	86 (85)	60 (97)	0.02
RRT, n (%)	1 (1)	1(1)	0	0.99
ECMO, n (%)	3 (2)	2 (2)	1 (2)	0.99
**Treatments**				
Surgical clipping, n (%)	33 (20)	15 (15)	18 (29)	0.04
Endovascular coiling, n (%)	110 (67)	67 (66)	43 (69)	0.74
Nimodipine (prophylaxis), n (%)	141 (87)	89 (88)	52 (84)	0.48
Osmotic therapy, n (%)	74 (45)	43 (43)	31 (50)	0.42
Induced Hypertension, n (%)	97 (60)	53 (53)	44 (71)	0.001
Barbituric coma, n (%)	34 (21)	14 (14)	20 (32)	0.009
Induced hypothermia, n (%)	30 (18)	9 (9)	21 (34)	0.001
Decompressive craniectomy, n (%)	15 (9)	6 (6)	9 (15)	0.09
Intra-arterial nimodipine, n (%)	53 (33)	20 (20)	33 (53)	0.001
Angioplasty, n (%)	23 (14)	12 (12)	11 (18)	0.36
**ICP/PbtO**_**2**_** guided-therapy**				0.001
No therapy	61 (37)	58 (57)	3 (5)	< 0.05
ICP/PbtO_2_ guided therapy	54 (33)	0	54 (87)	< 0.05
ICP only guided therapy	48 (29)	43 (43)	5 (8)	< 0.05
**Neurological complications**				
Seizures, n (%)	59 (36)	43 (43)	16 (26)	0.04
Rebleeding, n (%)	15 (9)	5 (5)	10 (16)	0.03
Hydrocephalus, n (%)	111 (68)	82 (81)	29 (47)	0.001
DCI, n (%)	59 (36)	30 (30)	29 (47)	0.03
Intracranial hypertension, n (%)	95 (58)	55 (55)	40 (65)	0.25
**Outcomes**				
ICU LOS, days (IQR)	16 (10–22)	15 (10–21)	18 (9–26)	0.20
Hospital LOS, days (IQR)	27 (13–52)	28 (13–49)	25 (10–54)	0.78
GOS, median (IQR)	3 (1–4)	3 (1–5)	2 (1–4)	0.15
Unfavorable outcome, n (%)	111 (68)	60 (59)	38 (61)	0.87
ICU death, n (%)	64 (39)	34 (34)	30 (48)	0.07
Hospital death, n (%)	68 (42)	38 (38)	30 (48)	0.19

Data are presented as count (%), mean ± SD or median (IQRs).

*N* number, *IQR* interquartile range, *APACHE* acute physiology and chronic health evaluation, *SOFA* sequential organ failure assessment, *GCS* Glasgow coma scale, *WFNS* world federation of neurological surgeons, *COPD* chronic obstructive pulmonary disease, *RRT* renal replacement therapy, *ECMO* extra-corporeal membrane oxygenation, *PbtO*_*2*_ brain tissue oxygenation, *DCI* delayed cerebral ischemia, *ICU* intensive care unit, *LOS* length of stay, *GOS* Glasgow outcome scale.

### ICP and ICP/PbtO_2_ monitoring

The characteristics of the two groups are shown in Table 1. Patients in the ICP/PbtO_2_ group underwent more frequently vasopressors or inotropic therapy and required more frequently invasive mechanical ventilation. Although the most used modality of treatment of the culprit aneurysm was endovascular coiling in the whole cohort, patients in the ICP/PbtO_2_ group presented more frequently with intraparenchymal hematoma and underwent more frequently surgical clipping than patients in the ICP group. The patients in the ICP/PbtO_2_ group developed more neurological complications such as re-bleeding and DCI than the other patients. Both ICU and hospital mortality were numerically higher, although not significantly different, in the ICP/PbtO_2_ group, while UO was similar in both groups.

Of the 62 patients in the ICP/PbtO_2_ group, brain hypoxia occurred in 54/62 (87%) patients and severe brain tissue hypoxia occurred in 39/62 (63%) patients. The overall burden of brain tissue hypoxia was 316.48 (102.32–560.89) mmHg*h. The burden of severe brain hypoxia was 36.88 (10.25–158.75) mmHg*h.

### Unfavorable neurological outcome and PbtO_2_ guided therapy

Patients with UO had higher severity scores on admission, received more frequently vasopressors and mechanical ventilation, were more often treated with surgical clipping and less frequently with prophylactic nimodipine. They also developed more complications (re-bleeding, intracranial hypertension and DCI; Supplemental Table S1). However, the proportion of patients receiving ICP/PbtO_2_ guided therapy (34/98, 35% vs. 20/65, 31%, *p* = 0.62) was similar between the two groups. In the multivariable analysis (Table 2) adjusted for age, poor grade on admission, the development of intracranial hypertension, DCI, presence of intraparenchymal hematoma, endovascular treatment, nimodipine prophylaxis, combined ICP/PbtO_2_-guided therapy (0.55 [0.20–1.46]) was not independently associated with UO (Supplemental Fig. 2).

**Table 2:** Logistic regression analysis to identify variables independently associated with 6-month unfavorable neurologic outcome.

	Univariable analysis OR (95% CI)	*p*-value	Multivariable analysis OR (95% CI)	*p*-value
Age	1.02 (1.00–1.05)	0.01	1.06 (1.02–1.01)	0.002
Poor grade (WFNS 4–5)	2.45 (1.16–5.16)	0.02	2.00 (0.77–5.23)	0.17
Intracranial hypertension	8.36 (4.09–17.09)	0.001	9.19 (3.87–21.82)	0.001
DCI	3.09 (1.52–6.31)	0.002	7.66 (2.71–21.69)	0.001
Endovascular treatment	0.62 (0.32–1.20)	0.29	0.95 (0.38–2.41)	0.79
Nimodipine prophylaxis	0.12 (0.03–0.55)	0.006	0.06 (0.01–0.35)	0.001
Intraparenchymal hematoma	2.83 (1.41–5.70)	0.004	3.32 (1.28–8.58)	0.009
ICP/PbtO_2_ guided therapy	2.83 (1.41–5.70)	0.81	0.55 (0.20–1.46)	0.10

Data are reported as odds ratio (OR) and 95% confidence intervals (CIs).

*WFNS* world federation of neurological surgeons, *ICP* intracranial hypertension, *PbtO*_*2*_ brain tissue oxygenation, *DCI* delayed cerebral ischemia.

### Hospital mortality and PbtO_2_ guided therapy

Non-survivors had higher severity scores on admission, suffered from often from chronic respiratory obstructive disease and cancer, received more frequently vasopressors and mechanical ventilation, were more often treated with surgical clipping, developed more complications (re-bleeding, hydrocephalus, intracranial hypertension and DCI) and underwent more specific therapies (osmotic therapy, barbituric coma and induced hypothermia) than survivors (Supplemental Table S1). However, the proportion of patients receiving PbtO_2_-guided therapy was similar between the two groups. In the Cox regression analysis adjusted for age, endovascular treatment, intracranial hypertension, DCI, intraparenchymal hematoma and nimodipine prophylaxis, combined ICP/PbtO_2_-guided therapy (Supplemental Table S2) was not independently associated with hospital mortality.

### ICP- versus ICP/PbtO_2_-guided therapy

Among the 102 patients that received a therapy based on invasive neuromonitoring (either ICP- only or ICP/PbtO_2-_guided therapy), 75 (74%) had UO (Supplemental Table S3). Patients with UO received less prophylactic nimodipine and were less treated with endovascular coiling; also, they also had more episodes of intracranial hypertension (Supplemental Table S4). In the multivariable analysis adjusted for endovascular treatment and nimodipine prophylaxis, PbtO_2_ guided therapy was associated with a lower risk of UO (OR 0.33 [95% CI 0.12–0.89]) in 6 months (Table 3, Supplemental Fig. 2). PbtO_2_-guided therapy remained associated with UO even when the APACHE II score or poor grade on admission (WFNS 4–5) were added to the multivariable models (Supplemental Table S5).

**Table 3:** Logistic regression analysis to identify possible association between combined ICP/PbtO_2_ guided therapy and 6-month unfavorable neurologic outcome in patients undergoing ICP- or ICP/PbtO_2_ guided-therapy (n = 102).

	Univariable analysis OR (95% CI)	*p*-value	Multivariable analysis OR (95% CI)	*p*-value
ICP/PbtO_2_ guided therapy	0.29 (0.11–0.77)	0.02	0.33 (0.12–0.89)	0.02
Nimodipine prophylaxis	0.14 (0.02–1.23)	0.07	0.23 (0.03–1.98)	0.12
Endovascular therapy	0.29 (0.09–0.93)	0.04	0.47 (0.15–1.49)	0.19

Data are reported as odds ratio (OR) and 95% confidence intervals (CIs).

*ICP* intracranial hypertension, *PbtO*_*2*_ brain tissue oxygenation.

In this subgroup of patients, hospital mortality was 56%; non-survivors had more frequently episodes of intracranial hypertension than others. In the Cox regression analysis, PbtO_2_-guided therapy (HR 0.70 [0.41–1.12]; Supplemental Table S6) was not associated with survival.

### Aneurysmal SAH

Among the 143 patients admitted with aSAH, 82 were monitored with ICP only and 61 with ICP and PbtO_2_; 42 patients received ICP-guided therapy and 53 patients received ICP/PbtO_2_-guided therapy*.* Combined ICP/PbtO_2_-guided therapy was not independently associated with UO nor with mortality; however, among patients receiving a therapy based on invasive neuromonitoring (ICP only or ICP/PbtO_2_-guided therapy), PbtO_2_-guided therapy was associated with a lower probability of UO in the multivariable models (Supplemental Tables S7, S8, S9, S10, S11, S12, S13, S14).

## Discussion

In this retrospective single-center cohort of patients with non-traumatic SAH, the use of ICP/PbtO_2_ guided therapy compared to patients that received no therapy or ICP only guide therapy was not associated with an improved outcome. Only in the subgroup of patients requiring a therapy driven by MMM (ICP or combined ICP/PbtO_2_), PbtO_2_-guided therapy was associated with a lower risk of UO than ICP-guided therapy.

MMM has been widely advocated to assess poor grade neurocritical patients, since the severity of the initial injury or the concomitant use of sedation and/or neuromuscular blockade significantly reduce the reliability of clinical examination to detect neurologic deterioration or tissue hypoxia^14^. PbtO_2_ monitoring provide focal but clinically relevant information on tissue oxygenation and, if adequately interpreted and included into a therapeutic protocol, could act as an early trigger to initiate therapies even in the presence of normal ICP values^17^. This is also relevant in SAH patients, as sustained and severe increase of ICP and tissue hypoxia can be driven by several mechanisms including the direct effect of the bleeding, cerebral swelling, diffuse hypoperfusion or delayed vasoconstriction^17^.

Brain oxygen values reflect an equilibrium between oxygen delivery (i.e. cerebral blood flow, hemoglobin and arterial oxygenation), consumption (i.e. brain metabolism, mitochondria and body temperature) and extraction (microcirculation and blood–brain barrier)^37,38^. In SAH patients, low PbtO_2_ has been associated with different pathologic pathways, such as low cerebral blood flow^30,39^, lung injury with hypoxemia^22,40^ and/or anemia^41^. As such, strategies aiming at increasing cerebral blood flow, using high inspired oxygen fraction on the ventilator or prescribing red blood cell transfusion can increases PbtO_2_ levels in some of these patients^42,43^. However, low PbtO_2_ levels do not necessarily represent tissue ischemia^37^ and some studies failed to show an association between low PbtO_2_ and unfavorable outcomes^43,44^. Future studies should evaluate in larger cohorts the optimal threshold of PbtO_2_ to predict poor neurological outcome and mortality and therefore optimize therapies in SAH patients. The integration of ICP/PbtO_2_ monitoring with other tools (i.e. electroencephalography, cerebral microdialysis) should therefore be considered as a useful MMM approach to precisely define the pathophysiology of brain injury and individualize clinical management in SAH patients, although additional data are necessary to understand its role on modifying patients’ outcome^14,45^.

In TBI patients, Okonkwo et al.^26^ showed that the use of PbtO_2_ guided therapy using a specific and complex protocol reduced the burden of brain hypoxia when compared to patients who underwent ICP guided therapy only. Furthermore, two meta-analysis reported that ICP/PbtO_2_ guided therapy was associated with improved neurologic outcome, when compared with standard ICP-guided therapy^46,47^; although large randomized trials in TBI patients are currently ongoing to provide more robust evidence. In SAH patients, the burden of brain hypoxia remains relatively high despite of protocolized PbtO_2_-guided therapy; in one study, Rass et al.^44^ showed that 81% of SAH patients included in two experienced centers had at least one episode of brain hypoxia (i.e.PbtO_2_ < 20 mmHg). This could explain why we could not find an association of PbtO_2_-guided therapy compared to no therapy and/or ICP-guided therapy with an improvement in neurological outcome, since the proposed treatment may not be enough to reverse tissue hypoxia, even in the presence of protocolized strategies. Moreover, we lack robust data showing which intervention (i.e. raising blood pressure, transfusions, changes in PaCO_2_ or body temperature etc.) is the most effective to correct brain hypoxia in SAH patients. Also, as brain hypoxia can occur either in the early phase but also after several days since admission because of DCI, the lack of adequate evidence supporting effective therapeutic strategies to treat DCI would also limit the effectiveness of PbtO_2_-guided therapies in this setting.

Some patients had normal ICP and PbtO_2_ values and required no intervention; moreover, as the monitoring per se cannot improve outcome alone since the decision to treat is ultimately at the clinician’s discretions we performed an additional analysis including only those patients where an intervention was undertaken, either guided by ICP alone or by ICP/PbtO_2_. In this subgroup of patients, PbtO_2_-guided therapy was associated with a favorable neurological outcome when compared to ICP-guided therapy. These results should be interpreted with caution as all patients in the ICP-guided therapy subgroup experienced intracranial hypertension, which is a well-known determinant of poor outcome in SAH patients, while only 61% had this complication in the ICP/PbtO_2_ group. Unfortunately, we could not assess the “intensity” (the highest ICP value) and “duration” of intracranial hypertension, which have both been shown to predict neurological outcome in this setting^48^. However, brain hypoxia is also a determinant of UO after SAH and deserves further attention in the management of these patients, as for intracranial hypertension. In a before/after study, Veldeman et al. showed that the implementation of PbtO_2_ and microdialysis monitoring in poor grade SAH patients was associated with an earlier detection of DCI and a significant reduction in the occurrence of UO, from 60 to 46%^49^. In another before/after study including good grade SAH patients with secondary deterioration, the introduction of invasive neuromonitoring (PbtO_2_ and microdialysis) was associated a significant reduction of silent cerebral infarctions, although no significant effects on neurological outcome was observed^50^. However, as the introduction of neuromonitoring could also been associated with other significant changes in diagnostic procedure and patients’ management (i.e. before and after study), it is difficult to conclude the effectiveness of invasive neuromonitoring on patients’ outcome from these studies.

Our study has some limitations. First, there could have been a selection bias, since we had a limited number of PbtO_2_ monitoring devices and the decision to monitor some patients might have been influenced by factors which are not collected in this study. Second, due to its retrospective design, some deviations from protocolized care or decisions to tolerate quite low PbtO_2_ values (i.e. 15–20 mmHg) in case of improvement of clinical status and/or awakening could not be adequately addressed. Also, all single therapeutic interventions and their effects on PbtO_2_ values over time were not specifically reported and we cannot exclude that the intensity of care and overall management were similar between groups, independently on PbtO_2_ monitoring. Prospective studies are required to assess these issues and provide relevant information on PbtO_2_ changes after different therapies. Third, the number of patients receiving PbtO_2_ monitoring was relatively limited, which may have reduced the power for future statistical adjustment to assess smaller effects of PbtO_2_ monitoring on patients’ outcome. Fourth, as this cohort reflected the experience of a single center, generalizability of our findings might be limited. Fifth, we did not specifically report all single therapeutic interventions and their effects on PbtO_2_ values over time. Finally, we included also non-aneurysmal non-traumatic SAH to the study cohort; although these patients have in general a better neurological outcome than those suffering from aneurysmal SAH, poor grade non-aneurysmal SAH still present a probability of UO exceeding 50%^51^.

## Conclusions

In this cohort of non-traumatic SAH patients ICP/PbtO2 monitoring was not associated with a better outcome. In a secondary analysis, which is hypothesis-generating, PbtO_2_-guided therapy was associated with better neurological recovery in the subgroup of patients requiring therapeutic interventions driven by neuromonitoring (ICP alone or ICP/PbtO_2_). Prospective studies are needed to properly assess the role of combined ICP/PbtO_2_ monitoring and PbtO_2_-guided therapy in SAH patients.

## Supplementary Information

Supplementary Information.

## Data Availability

Due to ethical restrictions, the datasets used and/or analyzed during the current study are available from the corresponding author on reasonable request. All data generated after the analysis during this study are included in this published article and its supplementary information files.
