# Supplementary material for: Brain tissue oxygenation guided therapy and outcome in non-traumatic subarachnoid hemorrhage
Source: Sci Rep. 2021 Aug 10;11:16235. doi: 10.1038/s41598-021-95602-6 (PMC8355344; doi:10.1038/s41598-021-95602-6)
Supplement: Supplementary file 1 — Supplementary Information. [file 41598_2021_95602_MOESM1_ESM.docx]

**Brain tissue oxygenation guided therapy and outcome in non-traumatic subarachnoid hemorrhage**

*Elisa GOUVEA BOGOSSIAN^1*^, Daniela DIAFERIA^1^, Narcisse NDIEUGNOU DJANGANG ^1^, Marco MENOZZI^1^, Jean-Louis VINCENT^1^, Marta TALAMONTI^1^, Olivier DEWITTE^2^, Lorenzo PELUSO^1^, Sami BARRIT ^2^, Mejdeddine AL BARAJRAJI^2^, Joachim ANDRE^3^, Sophie SCHUIND^2^, Jacques CRETEUR^1^, Fabio Silvio TACCONE^1^*

*^1^Department of Intensive Care*

*Erasme Hospital*

*Université Libre de Bruxelles*

*Route de Lennik, 808*

*1070 Brussels, Belgium*

*^2^Department of Neurosurgery*

*Erasme Hospital*

*Université Libre de Bruxelles*

*Route de Lennik, 808*

*1070 Brussels, Belgium*

*^3^Department of Radiology*

*Erasme Hospital*

*Université Libre de Bruxelles*

*Route de Lennik, 808*

*1070 Brussels, Belgium*

**Supplemental Material: Patient management**

Diagnostic work-up of SAH in our Department includes an initial cerebral CT-scan without contrast; if there is a high clinical suspicion of SAH but negative radiologic findings, a spinal tap is performed. If a SAH is present on the initial non-contrast CT, a CT angiogram (CTA) is performed to identify the origin of the bleeding. Digital subtraction angiography (DSA) is performed within 24 hours (or earlier if clinical symptoms are present for more than 3 days) and therapeutic intervention (i.e. endovascular coiling vs. surgical clipping) is determined in a multidisciplinary discussion including ICU physicians, neurosurgeons and neuroradiologists, considering cerebrovascular anatomy, aneurysm location, size and shape and the presence of intra-parenchymal hematomas. Angio-Magnetic Resonance Imaging (MRI) is performed when no lesion is identified on angiography. If no aneurysm is identified, a new DSA is performed after 7-14 days of the initial bleed, according to the severity of bleeding. If again no aneurysm is identified a DSA, CTA or angio-MRI is repeated at 1 month .

All patients are monitored using a central venous catheter and an arterial line; if the GCS score on admission is <9, the patient's trachea is intubated to protect the airways. In comatose patients and in patients with rapid deterioration of neurological status and a cerebral CT scan suggesting elevated intracranial pressure (ICP) or hydrocephalus (defined by CT scan with Evans index > 0.3 ) an external ventricular drain (EVD) is implanted. All patients undergo frequent neurologic evaluation (every 2 hours) and use of sedatives and analgesics is minimized. No antiepileptic prophylaxis is routinely given but continuous electroencephalography (EEG) monitoring is initiated as soon as possible in comatose patients and those with significant fluctuations of conscious level, in order to exclude seizures. Oral nimodipine (60 mg q4h) is administered routinely, except in the presence of hypotension requiring vasopressor therapy. Before aneurysm treatment, mean arterial pressure (MAP) is tightly controlled (<105 mmHg) with intravenous β-blockers (labetalol or metoprolol) or calcium channel blockers (nicardipine); thereafter a higher MAP (up to 130 mmHg) is generally tolerated. In the patients with ICP monitoring, intracranial hypertension defined as a prolonged increase in ICP (typically >20 mmHg for more than 5 min) is treated with the head of the bed elevated at 30°, intravenous mannitol and CSF drainage. Administration of barbiturates or induction of hypothermia (32-34°C) is initiated if there is no response to the initial therapeutic interventions. Red Blood Cell transfusion is given if the hemoglobin level decreases to <7 g/dL, although higher thresholds are considered in more severely ill patients and those with cerebral vasospasm. In addition to echocardiography, invasive hemodynamic monitoring (PiCCO, Pulsion, Munich, Germany) is placed in patients requiring vasopressors and/or with increased lactate levels with a poor response to fluid therapy and/or development of respiratory failure. A local insulin protocol is applied to keep blood glucose levels between 110 and 150 mg/dL. The presence of “cerebral vasospasm” is investigated by daily transcranial Doppler; in the presence of high flow intra-cranial artery velocities (>200 cm/sec) and/or high clinical suspicion, the diagnosis is confirmed by angio-CT and angiography.

DCI s defined as: a) the occurrence of focal neurological impairment; b) a decrease of at least 2 points on the Glasgow Coma Scale that last for at least 1 hour that is not associated with aneurysm occlusion or other causes such as brain edema, hydrocephalus, rebleeding, seizures, metabolic and systemic disturbances; c) cerebral infarction shown on CT-scan that is not related to invasive procedures and without other explanation. In patients where clinical evaluation is compromised (i.e. GCS<9 or sedated), the presence of cerebral hypoperfusion diagnosed on perfusion CT-scan (which is decided according to neuromonitoring findings, including continuous EEG, PbtO_2_ and, more recently, microdialysis) is also considered as DCI.

Aggressive hypertensive therapy with vasopressors (i.e. norepinephrine, which is titrated to raise MAP by 15 mmHg every 10 minutes to a maximum of 130 mmHg) is therefore initiated; if the response to this intervention is unsatisfactory, therapeutic options include intra-arterial vasodilators (i.e. nimodipine or milrinone administered locally during angiography) and/or trans-luminal balloon angioplasty (i.e. proximal middle cerebral artery vasospasm), in case of localized vasospasm, or intra-arterial vasodilators and inotropic agents (i.e. dobutamine or milrinone to obtain a cardiac index >4.0 L/min.m^2^), in case of diffuse vasospasm. In refractory cases, an intra-carotid catheter with continuous vasodilators administrations may be also used.

No antibiotic prophylaxis for the insertion of external ventricular devices, invasive neuromonitoring (PbtO_2_ and microdialysis catheters), endovascular coiling and surgical clipping of the aneurysm is administered.

Decisions to withdraw life-support are made after interdisciplinary discussions based on various elements, including persistent deep coma, refractory status epilepticus, uncontrolled intracranial hypertension and the extent of brain damage on cerebral CT and MRI.

**Supplemental Figure 1A:** Flow-chart of the protocolized intracranial pressure guided therapy in patients suffering from subarachnoid hemorrhage (SAH).

**
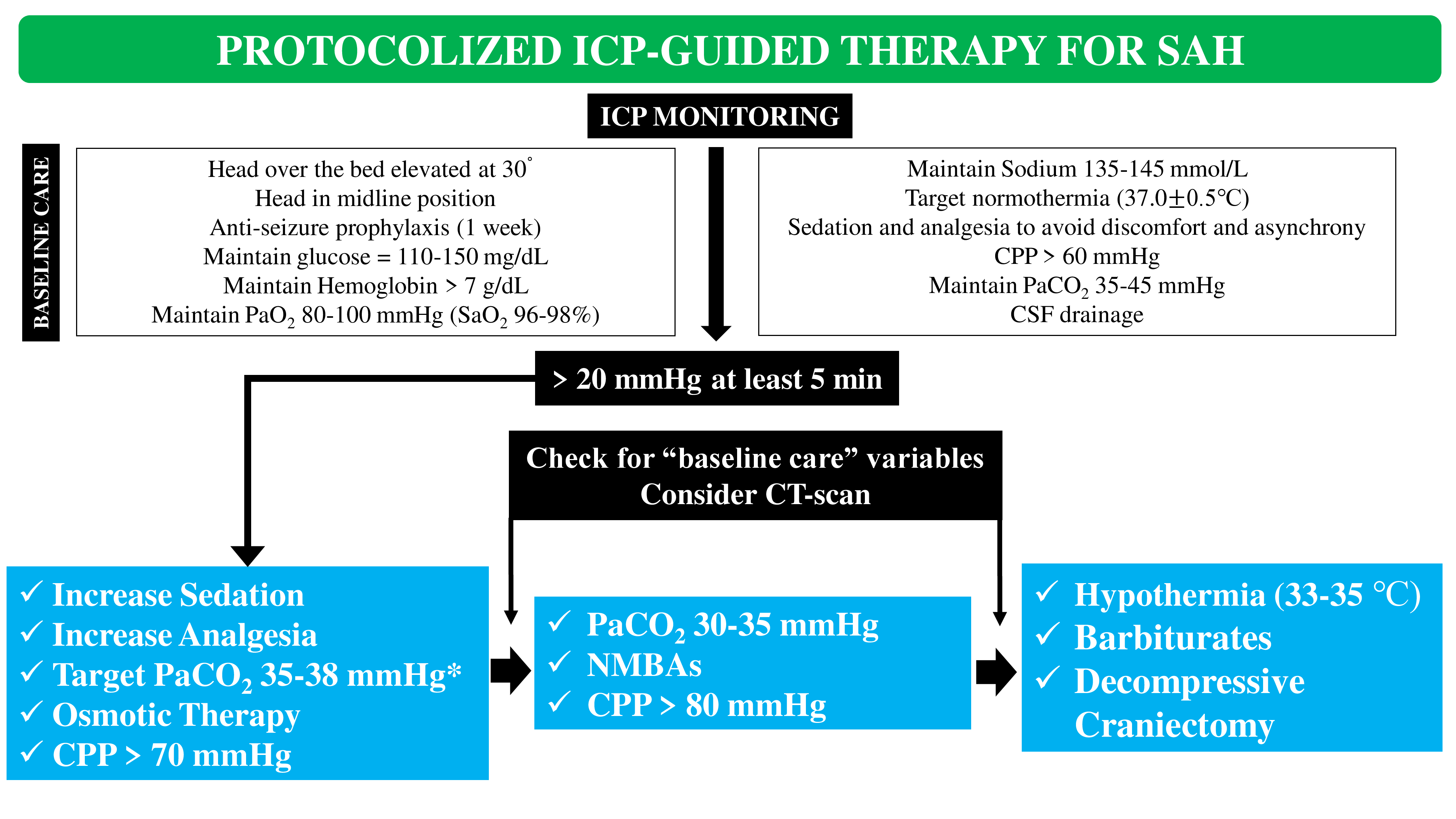
**

*if not already within these ranges

CPP = cerebral perfusion pressure; CSF = cerebrospinal fluid; NMBAs = neuro-muscular blocking agents

**Supplemental Figure 1B:** Flow-chart of the protocolized brain oxygen (PbtO_2_) guided therapy in patients suffering from subarachnoid hemorrhage (SAH).


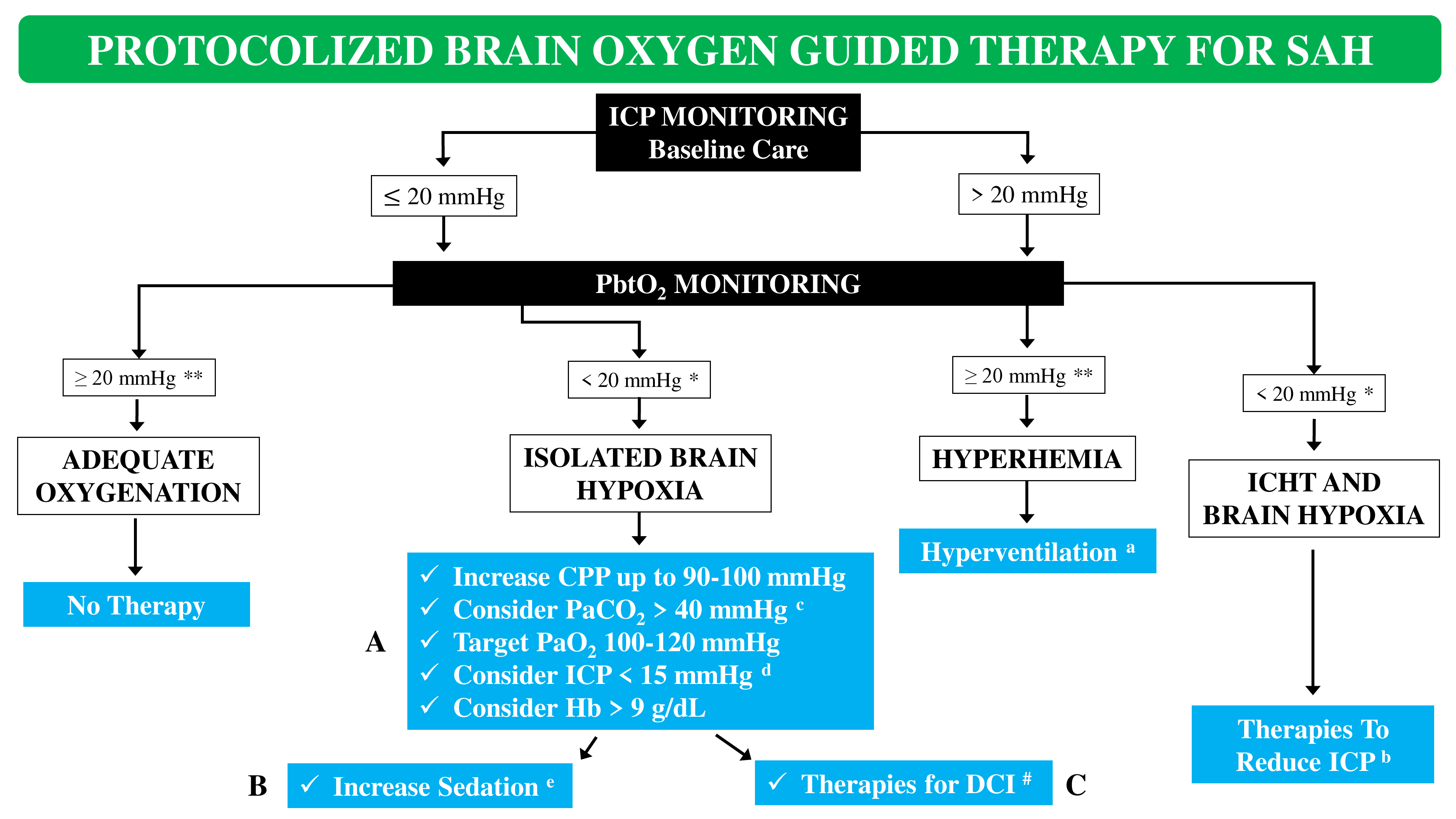


ICHT = intracranial hypertension; ICP = intracranial pressure; CPP = cerebral perfusion pressure; Hb = hemoglobin

*check for PaO_2_/SaO_2_ to exclude hypoxemia

**check for PaO_2_ to exclude values > 120 mmHg

The order of which intervention to be started first is dependent on patients’ condition and treating physician

A = Suspected low oxygen delivery

^a^ if ICP is between 20-25 mmHg and patient is awakening, no specific therapy could be considered.

^b^ see Supplemental Figure 1A

^c^ if ICP remains below 20 mmHg

^d^ if ICP is between 15 and 20 mmHg with tissue hypoxia

B = Suspected increased oxygen consumption (i.e. agitation; seizures; inadequate sedation on EEG monitoring)

C = Suspected delayed cerebral ischemia (DCI): diagnosis of DCI is based on a combination of parameters (i.e. clinical deterioration when clinical examination is available or EEG monitoring, PbtO_2_ and trans-cranial Doppler for unconscious patients) and the demonstration of altered cerebral perfusion on CT-perfusion $\pm$narrowing of intracranial vessels (angiography or angio-CT scan).

^#^These includes: a) induced hypertension; b) intra-arterial vasodilators; c) cerebral angioplasty; d) rescue therapies (i.e. intra-carotid catheter for continuous infusion of vasodilators; therapeutic hypothermia; steroids; high-dose magnesium)

**Supplemental Figure 2:** Distribution of the Glasgow Outcome Scale (GOS) between ICP monitored vs ICP-PbtO2 monitored groups and ICP-guided vs. ICP-PbtO_2_ guided therapy groups.

GOS: Glasgow outcome scale; ICP: intracranial pressure; PbtO2: brain tissue oxygenation

**Supplemental Table S1:** Characteristics of the study population, according to mortality and neurological outcome. Data are presented as count (%), mean ±SD or median (IQRs).

|  | **Survivors**  **N=95** | **Non-survivors**  **N=68** | **p value** | **FO**  **(N=65)** | **UO**  **(N=98)** | **p value** |
| --- | --- | --- | --- | --- | --- | --- |
| Age, years | 54 (±11) | 56 (±15) | 0.34 | 53 (45-60) | 56 (47-67) | 0.08 |
| Male gender, n (%) | 43 (45) | 23 (34) | 0.15 | 32 (49) | 34 (35) | 0.07 |
| APACHE on admission | 17 (12-21) | 20 (15-23) | 0.008 | 16 (11-19) | 20 (15-23) | 0.001 |
| SOFA on admission | 6 (4-9) | 9 (5-10) | 0.02 | 6 (3-8) | 9 (5-10) | 0.001 |
| GCS on admission | 6 (3-13) | 3 (3-10) | 0.09 | 7 (3-13) | 5 (3-8) | 0.01 |
| Poor grade (WFNS 4-5), n (%) | 71 (75) | 55 (81) | 0.45 | 44 (68) | 82 (84) | 0.02 |
| High risk modified Fisher scale, n (%) | 91 (96) | 63 (93) | 0.49 | 63 (97) | 91 (93) | 0.32 |
| Ruptured Aneurysm, n (%) | 81 (85) | 62 (91) | 0.34 | 56 (86) | 87 (89) | 0.63 |
| Anterior location aneurysm, n (%) | 60 (63) | 45 (66) | 0.74 | 39 (60) | 66 (67) | 0.40 |
| Intraparenchymal hematoma, n (%) | 30 (32) | 30 (44) | 0.14 | 15 (23) | 45 (46) | 0.005 |
| Hypertension, n (%) | 51 (54) | 29 (43) | 0.20 | 31 (48) | 49 (50) | 0.87 |
| Diabetes mellitus, n (%) | 12 (13) | 6 (9) | 0.61 | 9 (14) | 9 (9) | 0.45 |
| Heart disease, n (%) | 10 (11) | 8 (12) | 0.81 | 5 (8) | 13 (13) | 0.32 |
| Previous neurological disease, n (%) | 9 (10) | 8 (12) | 0.80 | 8 (12) | 19 (9) | 0.60 |
| Chronic Kidney Disease, n (%) | 3 (3) | 1 (2) | 0.64 | 2 (3) | 2 (2) | 0.99 |
| COPD, n (%) | 4 (4) | 13 (19) | 0.003 | 4 (6) | 13 (13) | 0.19 |
| Immunosuppression, n (%) | 5 (5) | 4 (6) | 0.99 | 2 (3) | 7 (7) | 0.32 |
| Cancer, n (%) | 3 (3) | 8 (12) | 0.05 | 3 (5) | 8 (8) | 0.53 |
| Cirrhosis, n (%) | 3 (3) | 4 (6) | 0.45 | 1 (2) | 6 (6) | 0.25 |
| Vasopressors, n (%) | 63 (66) | 61 (90) | 0.001 | 40 (62) | 82 (84) | 0.001 |
| Inotropic agents, n (%) | 21 (22) | 24 (36) | 0.08 | 14 (22) | 31 (32) | 0.21 |
| Mechanical ventilation, n (%) | 78 (82) | 68 (100) | 0.001 | 52 (80) | 94 (96) | 0.002 |
| RRT, n (%) | 0 | 1 (2) | 0.42 | 0 | 1 (1) | 0.99 |
| ECMO, n (%) | 0 | 3 (4) | 0.07 | 0 | 3 (3) | 0.28 |
| Endovascular treatment (coiling), n (%) | 65 (68) | 45 (66) | 0.87 | 47 (72) | 63 (98) | 0.31 |
| Surgical treatment (clipping), n (%) | 14 (15) | 19 (28) | 0.05 | 5 (8) | 28 (29) | 0.001 |
| Nimodipine (prophylaxis), n (%) | 86 (91) | 55 (81) | 0.10 | 63 (97) | 78 (80) | 0.001 |
| ICP only guided therapy | 18 (19) | 30 (44) | 0.001 | 7 (11) | 41 (42) | 0.001 |
| ICP/PbtO_2_ guided therapy | 28 (30) | 26 (38) | 0.31 | 20 (31) | 34 (35) | 0.62 |
| Seizures, n (%) | 35 (37) | 24 (35) | 0.87 | 20 (31) | 39 (40) | 0.25 |
| Re-bleeding, n (%) | 3 (3) | 12 (18) | 0.002 | 1 (2) | 14 (14) | 0.005 |
| Hydrocephalus, n (%) | 71 (75) | 40 (59) | 0.04 | 49 (75) | 62 (63) | 0.12 |
| DCI, n (%) | 27 (28) | 32 (47) | 0.02 | 14 (22) | 45 (46) | 0.002 |
| Intracranial hypertension, n (%) | 38 (40) | 57 (84) | 0.001 | 19 (29) | 76 (78) | 0.001 |
| Induced Hypertension, n (%) | 59 (62) | 38 (56) | 0.52 | 40 (62) | 57 (58) | 0.75 |
| Osmotic therapy, n (%) | 26 (27) | 48 (71) | 0.001 | 11 (17) | 63 (64) | 0.001 |
| Barbituric coma, n (%) | 9 (10) | 25 (37) | 0.001 | 4 (6) | 30 (31) | 0.001 |
| Hypothermia, n (%) | 8 (8) | 22 (32) | 0.001 | 4 (6) | 26 (27) | 0.001 |
| Decompressive craniectomy, n (%) | 9 (10) | 6 (9) | 0.99 | 4 (6) | 11 (11) | 0.41 |
| Intra-arterial nimodipine, n (%) | 31 (33) | 22 (32) | 0.99 | 21 (32) | 32 (33) | 0.99 |
| Cerebral angioplasty, n (%) | 15 (16) | 8 (12) | 0.50 | 10 (15) | 13 (13) | 0.84 |
| ICU LOS, days | 19 (15-27) | 10 (5-15) | 0.001 | 18 (14-23) | 14 (6-21) | 0.001 |
| Hospital LOS, days | 45 (29-85) | 11 (5-16) | 0.001 | 40 (27-70) | 15 (7-38) | 0.001 |

APACHE : acute physiology and chronic health evaluation; SOFA : sequential organ failure assessment; GCS: Glasgow coma scale WFNS: world federation of neurological surgeons; COPD: chronic obstructive pulmonary disease; RRT: renal replacement therapy; ECMO: extra-corporeal membrane oxygenation. PbtO_2_: brain tissue oxygenation; DCI: delayed cerebral ischemia; ICHT: Intracranial hypertension; ICU: intensive care unit; LOS: length of stay.

**Supplemental Table S2:** Cox regression to identify variables independently associated with hospital mortality. Data are reported as hazard ratio (HR) and 95 % confidence intervals (CIs).

|  | **Univariable analysis**  **HR (95% CI)** | **P value** | **Multivariable analysis**  **HR (95% CI)** | **P value** |
| --- | --- | --- | --- | --- |
| **Age** | 1.009 (0.99-1.03) | 0.37 | 1.01 (0.99-1.03) | 0.36 |
| **Nimodipine** | 0.51 (0.28-0.94) | 0.03 | 0.60 (0.32-1.12) | 0.11 |
| **Endovascular treatment** | 0.84 (0.51-1.39) | 0.50 | 0.80 (0.47-1.35) | 0.40 |
| **Intracranial hypertension** | 5.37 (2.81-10.26) | 0.0001 | 5.09 (2.62-9.87) | 0.001 |
| **DCI** | 1.51 (0.94-2.44) | 0.09 | 1.17 (0.72-1.91) | 0.53 |
| **Intraparenchymal hematoma** | 1.49 (0.93-2.41) | 0.10 | 1.07 (0.65-1.78) | 0.78 |
| **ICP/PbtO_2_ guided therapy** | 1.23 (0.75-2.00) | 0.23 | 1.28 (0.78-2.11) | 0.32 |

ICP: intracranial pressure; PbtO_2_: brain tissue oxygenation

**Supplemental table S3:** Characteristics of the patients undergoing ICP treatment or ICP/PbtO_2_ treatment. Data are presented as count (%), mean ±SD or median (IQRs).

|  | **All treated patients (N=102)** | **ICP**  **(N=48)** | **ICP/PbtO_2_**  **(N=54)** | **p value** |
| --- | --- | --- | --- | --- |
| **On Admission** | | | | |
| Age, mean (±SD) | 54 (±13) | 55 (±14) | 54 (±13) | 0.59 |
| Male gender, n (%) | 38 (37) | 16 (33) | 22 (41) | 0.54 |
| APACHE score, median (IQR) | 19 (14-22) | 20 (16-22) | 18 (13-21) | 0.07 |
| SOFA score, median (IQR) | 8 (5-10) | 8 (5-10) | 8 (3-10) | 0.22 |
| GCS, median (IQR) | 5 (3-11) | 5 (3-7) | 6 (3-13) | 0.32 |
| WFNS 4-5, n (%) | 81 (79) | 42 (88) | 39 (72) | 0.09 |
| mFisher scale 3 or 4 points, n (%) | 94 (92) | 47 (98) | 47 (87) | 0.06 |
| Intraparenchymal hematoma, n (%) | 48 (47) | 22 (46) | 26 (48) | 0.85 |
| Anterior circulation aneurysm, n (%) | 63 (62) | 39 (81) | 24 (44) | 0.001 |
| **Treatments** | | | | |
| Surgical clipping, n (%) | 26 (26) | 13 (27) | 13 (24) | 0.82 |
| Endovascular coiling, n (%) | 70 (69) | 29 (60) | 41 (76) | 0.13 |
| Nimodipine (prophylaxis), n (%) | 85 (83) | 38 (80) | 47 (87) | 0.30 |
| Osmotic therapy , n (%) | 74 (73) | 48 (100) | 26 (48) | 0.001 |
| Induced Hypertension, n (%) | 66 (65) | 22 (46) | 44 (82) | 0.001 |
| Barbituric coma, n (%) | 34 (33) | 17 (35) | 17 (32) | 0.68 |
| Induced hypothermia, n (%) | 29 (28) | 11 (23) | 18 (33) | 0.28 |
| Decompressive craniectomy, n (%) | 15 (15) | 7 (15) | 8 (15) | 0.99 |
| Intra-arterial nimodipine, n (%) | 43 (42) | 10 (21) | 33 (61) | 0.001 |
| Angioplasty, n (%) | 18 (18) | 7 (15) | 11 (20) | 0.60 |
| **Neurological complications** | | | | |
| Seizures, n (%) | 34 (33) | 19 (40) | 15 (28) | 0.22 |
| Rebleeding, n (%) | 13 (13) | 5 (10) | 8 (15) | 0.56 |
| Hydrocephalus, n (%) | 60 (59) | 35 (73) | 25 (46) | 0.009 |
| DCI, n (%) | 46 (45) | 17 (35) | 29 (54) | 0.08 |
| Intracranial hypertension, n (%) | 83 (81) | 48 (100) | 35 (65) | 0.001 |
| **Outcomes** | | | | |
| ICU LOS- days (IQR) | 16 (8-25) | 13 (6-23) | 18 (12-29) | 0.01 |
| Hospital LOS- days (IQR) | 20 (9-50) | 15 (16-39) | 27 (14-53) | 0.07 |
| GOS -points, median (IQR) | 1 (1-4) | 1 (1-3) | 2 (1- 4) | 0.06 |
| Unfavorable outcome, n (%)* | 75 (74) | 41 (85) | 34 (63) | 0.01 |
| ICU death, n (%) | 56 (55) | 30 (63) | 26 (48) | 0.17 |
| Hospital death, n (%) | 56 (55) | 30 (63) | 26 (48) | 0.17 |

**Supplemental Table S4 :** Characteristics of the patients undergoing ICP- or ICP/PbtO_2_ guided-therapy, according to hospital mortality and neurological outcome. Data are presented as count (%), mean ±SD or median (IQRs).

|  | **Survivors**  **N=46** | **Non-survivors**  **N=56** | **p value** | **FO**  **N=27** | **UO**  **N=75** | **p value** |
| --- | --- | --- | --- | --- | --- | --- |
| Age, years, mean (±SD) | 54 (±11) | 55 (±15) | 0.81 | 52 (±11) | 55 (±14) | 0.20 |
| Male gender, n (%) | 18 (39) | 20 (36) | 0.84 | 12 (44) | 36 (35) | 0.49 |
| APACHE on admission, median (IQR) | 18 (13-21) | 20 (15-22) | 0.09 | 18 (12-20) | 20 (15-22) | 0.03 |
| SOFA on admission, median (IQR) | 8 (5-10) | 9 (5-10) | 0.67 | 6 (5-10) | 9 (5-10) | 0.24 |
| GCS on admission, median (IQR) | 6 (3-13) | 3 (3-10) | 0.25 | 7 (3-13) | 5 (3-8) | 0.23 |
| Poor grade (WFNS 4-5), n (%) | 35 (76) | 46 (82) | 0.47 | 18 (67) | 63 (84) | 0.09 |
| High risk modified Fisher scale, n (%) | 43 (94) | 51 (91) | 0.73 | 26 (96) | 68 (91) | 0.68 |
| Aneurysm, n (%) | 43 (94) | 53 (93) | 0.99 | 26 (96) | 69 (92) | 0.67 |
| Anterior location aneurysm, n (%) | 28 (61) | 35 (63) | 0.99 | 14 (52) | 49 (65) | 0.25 |
| Intraparenchymal hematoma, n (%) | 20 (44) | 28 (50) | 0.55 | 9 (33) | 39 (52) | 0.12 |
| Hypertension, n (%) | 24 (52) | 21 (38) | 0.16 | 12 (44) | 33 (44) | 0.99 |
| Diabetes mellitus, n (%) | 6 (13) | 4 (7) | 0.34 | 4 (15) | 6 (8) | 0.45 |
| Heart disease, n (%) | 5 (11) | 7 (13) | 0.99 | 2 (7) | 10 (13) | 0.51 |
| Previous neurological disease, n (%) | 4 (9) | 6 (11) | 0.99 | 4 (15) | 6 (8) | 0.45 |
| Chronic Kidney Disease, n (%) | 0 | 1 (2) | 0.99 | 0 | 1 (1) | 0.99 |
| COPD, n (%) | 2 (4) | 9 (16) | 0.11 | 2 (7) | 9 (12) | 0.73 |
| Immunosuppression, n (%) | 2 (4) | 4 (7) | 0.68 | 0 | 1 (1) | 0.34 |
| Cancer, n (%) | 1 (2) | 5 (9) | 0.22 | 1 (4) | 5 (7) | 0.99 |
| Cirrhosis, n (%) | 2 (4) | 3 (5) | 0.99 | 0 | 5 (7) | 0.32 |
| Vasopressors, n (%) | 39 (85) | 52 (93) | 0.22 | 24 (89) | 67 (89) | 0.99 |
| Inotropic agents, n (%) | 19 (41) | 21 (38) | 0.84 | 13 (48) | 27 (36) | 0.36 |
| Mechanical ventilation, n (%) | 42 (91) | 56 (100) | 0.04 | 25 (93) | 73 (97) | 0.29 |
| RRT, n (%) | 0 | 1 (2) | 0.99 | 0 | 1 (1) | 0.99 |
| ECMO, n (%) | 0 | 2 (4) | 0.50 | 0 | 2 (3) | 0.99 |
| Endovascular treatment (coiling), n (%) | 34 (74) | 36 (64) | 0.40 | 22 (85) | 47 (63) | 0.03 |
| Surgical treatment (clipping), n (%) | 8 (17) | 18 (32) | 0.11 | 5 (8) | 28 (29) | 0.001 |
| Nimodipine (prophylaxis), n (%) | 40 (87) | 45 (80) | 0.43 | 26 (96) | 59 (79) | 0.04 |
| PbtO_2_ guided therapy, n (%) | 28 (61) | 26 (46) | 0.17 | 20 (74) | 34 (45) | 0.02 |
| Seizures, n (%) | 17 (37) | 17 (30) | 0.53 | 8 (30) | 26 (35) | 0.82 |
| Re-bleeding, n (%) | 3 (7) | 10 (18) | 0.14 | 1 (4) | 12 (16) | 0.18 |
| Hydrocephalus, n (%) | 28 (61) | 32 (57) | 0.84 | 15 (56) | 45 (60) | 0.82 |
| DCI, n (%) | 20 (44) | 26 (46) | 0.84 | 10 (37) | 36 (48) | 0.38 |
| Intracranial Hypertension, n (%) | 29 (63) | 52 (93) | 0.001 | 14 (52) | 67 (89) | 0.001 |
| Induced Hypertension, n (%) | 35 (76) | 31 (55) | 0.04 | 21 (78) | 45 (60) | 0.11 |
| Osmotic therapy, n (%) | 26 (57) | 48 (86) | 0.002 | 11 (41) | 63 (84) | 0.001 |
| Barbituric coma, n (%) | 9 (20) | 25 (45) | 0.01 | 4 (15) | 30 (40) | 0.02 |
| Hypothermia, n (%) | 8 (17) | 21 (38) | 0.03 | 4 (15) | 25 (33) | 0.08 |
| Decompressive craniectomy, n (%) | 9 (20) | 6 (11) | 0.27 | 4 (15) | 11 (15) | 0.99 |
| Intra-arterial nimodipine, n (%) | 25 (54) | 18 (32) | 0.03 | 17 (63) | 26 (35) | 0.01 |
| Cerebral angioplasty, n (%) | 13 (28) | 5 (9) | 0.02 | 8 (30) | 10 (13) | 0.08 |
| ICU LOS, days | 26 (19-34) | 10 (5-15) | 0.001 | 26 (18-30) | 13 (6-21) | 0.001 |
| Hospital LOS, days | 53 (34-92) | 10 (5-16) | 0.001 | 50 (33-84) | 14 (6-29) | 0.001 |

APACHE : acute physiology and chronic health evaluation; SOFA : sequential organ failure assessment; GCS: Glasgow coma scale WFNS: world federation of neurological surgeons; COPD: chronic obstructive pulmonary disease; RRT: renal replacement therapy; ECMO: extra-corporeal membrane oxygenation. PbtO_2_: brain tissue oxygenation; DCI: delayed cerebral ischemia; ICHT: Intracranial hypertension; ICU: intensive care unit; LOS: length of stay.

**Supplemental Table S5:** Logistic regression analysis to identify possible association between combined ICP/PbtO_2_ guided therapy and 6-month unfavorable neurologic outcome in patients undergoing ICP- or ICP/PbtO_2_ guided-therapy (n=102). Data are reported as odds ratio (OR) and 95 % confidence intervals (CIs).

|  | **Univariable analysis**  **OR (95% CI)** | **P value** | **Multivariable analysis**  **OR (95% CI)** | **P value** |  | **Univariable analysis**  **OR (95% CI)** | **P value** | **Multivariable analysis**  **OR (95% CI)** | **p-value** |
| --- | --- | --- | --- | --- | --- | --- | --- | --- | --- |
| **ICP/PbtO_2_ guided therapy** | 0.29 (0.11-0.77) | 0.02 | 0.36 (0.13-0.99) | 0.04 | **ICP/PbtO_2_ guided therapy** | 0.29 (0.11-0.77) | 0.02 | 0.35 (0.13-0.96) | 0.04 |
| **Nimodipine prophylaxis** | 0.14 (0.02-1.23) | 0.07 | 0.23 (0.03-1.92) | 0.16 | **Nimodipine prophylaxis** | 0.14 (0.02-1.23) | 0.07 | 0.22 (0.03-1.91) | 0.14 |
| **Endovascular therapy** | 0.29 (0.09-0.93) | 0.04 | 0.45 (0.13-1.53) | 0.23 | **Endovascular therapy** | 0.31 (0.10-0.89) | 0.04 | 0.45 (0.13-1.55) | 0.22 |
| **APACHE score** | 1.09 (1.01-1.18) | 0.03 | 1.06 (0.97-1.15) | 0.16 | **WFNS 4-5** | 2.63 (0.96-7.21) | 0.06 | 1.79 (0.61-5.23) | 0.33 |

APACHE : acute physiology and chronic health evaluation; WFNS: world federation of neurological surgeons; PbtO_2_: brain tissue oxygenation; ICP = intracranial pressure

**Supplemental Table S6:** Cox regression to identify variables independently associated with hospital mortality in patients undergoing ICP- or PbtO_2_ guided-therapy (n=102). Data are presented as hazard ratio (HR) and 95% confidence intervals (CI).

|  | **Univariable analysis**  **HR ( 95% CI)** | **P value** | **Multivariable analysis**  **HR (95% CI)** | **P value** |
| --- | --- | --- | --- | --- |
| **COPD** | 2.28 (1.11-4.69) | 0.03 | 2.59 (1.22-5.53) | 0.01 |
| **APACHE score** | 1.07 (1.08-1.12) | 0.02 | 1.06 (1.01-1.12) | 0.02 |
| **ICP/PbtO_2_ guided therapy** | 0.62 (0.37-1.06) | 0.33 | 0.70 (0.41-1.12) | 0.52 |
| **Surgical treatment** | 1.91 (1.09-3.34) | 0.03 | 1.75 (0.96-3.19) | 0.06 |
| **Rebleeding** | 2.42 (1.22-4.84) | 0.01 | 2.19 (1.06-4.53) | 0.04 |

ICP: intracranial hypertension; PbtO_2_: brain tissue oxygenation; COPD: chronic obstructive pulmonary disease; APACHE: acute physiology and chronic health evaluation

**Table S7:** Characteristics of the study population including only aneurysmal SAH (N=143), according to the type of neuro-monitoring. Data are presented as count (%), mean ±SD or median (IQRs).

|  | **All patients (N=143)** | **ICP**  **(N=82)** | **ICP/PbtO_2_**  **(N=61)** | **p value** |
| --- | --- | --- | --- | --- |
| **On Admission** | | | | |
| Age, mean (±SD) | 54 (±13) | 55 (±13) | 54 (±12) | 0.73 |
| Male gender, n (%) | 54 (38) | 30 (37) | 24 (39) | 0.86 |
| APACHE score, median (IQR) | 18 (13-21) | 18 (13-21) | 19 (13-22) | 0.62 |
| SOFA score, median (IQR) | 7 (4-10) | 6 (4-9) | 8 (4-10) | 0.35 |
| GCS, median (IQR) | 6 (3-12) | 6 (3-11) | 3 (3-13) | 0.52 |
| WFNS 4-5, n (%) | 110 (77) | 67 (79) | 45 (74) | 0.55 |
| mFisher scale 3 or 4 points, n (%) | 135 (94) | 80 (98) | 55 (90) | 0.07 |
| Intraparenchymal hematoma, n (%) | 57 (40) | 28 (34) | 29 (48) | 0.12 |
| Anterior circulation aneurysm, n (%) | 105 (73) | 77 (94) | 28 (46) | 0.001 |
| **Comorbidities** | | | | |
| HAS, n (%) | 70 (49) | 45 (55) | 25 (41) | 0.13 |
| DM, n (%) | 14 (10) | 9 (11) | 5 (8) | 0.78 |
| Heart disease, n (%) | 15 (11) | 10 (12) | 5 (8) | 0.58 |
| Previous neuro disease, n (%) | 14 (10) | 11 (13) | 3 (5) | 0.15 |
| CKD, n (%) | 3 (2) | 2 (2) | 1 (2) | 0.99 |
| Asthma /COPD, n (%) | 15 (11) | 9 (11) | 6 (10) | 0.99 |
| Immunosuppression, n (%) | 6 (4) | 5 (6) | 1 (2) | 0.24 |
| Cancer, n(%) | 7 (5) | 5 (6) | 2 (3) | 0.70 |
| Cirrhosis, n(%) | 5 (4) | 4 (5) | 1 (2) | 0.39 |
| **Support therapies during ICU stay** | | | | |
| Vasopressor, n(%) | 109 (76) | 52 (63) | 57 (93) | 0.001 |
| Inotropic, n(%) | 45 (32) | 9 (11) | 36 (59) | 0.001 |
| Mechanical ventilation, n(%) | 129 (90) | 70 (85) | 59 (97) | 0.03 |
| RRT, n (%) | 0 | 0 | 0 | - |
| ECMO, n (%) | 2 (1) | 1 (1) | 1 (2) | 0.99 |
| **Treatments** | | | | |
| Surgical clipping, n (%) | 33 (23) | 15 (18) | 18 (30) | 0.16 |
| Endovascular coiling, n (%) | 110 (77) | 67 (82) | 43 (71) | 0.16 |
| Nimodipine (prophylaxis), n (%) | 125 (87) | 74 (90) | 51 (84) | 0.31 |
| Osmotic therapy , n (%) | 68 (48) | 37 (45) | 31 (51) | 0.61 |
| Induced Hypertension, n (%) | 88 (62) | 45 (55) | 43 (71) | 0.08 |
| Barbituric coma, n (%) | 34 (24) | 14 (17) | 20 (33) | 0.05 |
| Induced hypothermia, n (%) | 30 (21) | 9 (11) | 21 (34) | 0.001 |
| Decompressive craniectomy, n (%) | 13 (9) | 4 (5) | 9 (15) | 0.07 |
| Intra-arterial nimodipine, n (%) | 52 (36) | 20 (24) | 32 (53) | 0.001 |
| Angioplasty, n (%) | 21 (15) | 10 (12) | 11 (18) | 0.35 |
| **ICP/PbtO_2_ Guided-therapy** | | | | 0.001 |
| No therapy | 48 (34) | 45 (55) | 3 (5) | <0.05 |
| ICP/PbtO_2_ guided therapy | 53 (37) | 0 | 53 (87) | <0.05 |
| ICP only guided therapy | 42 (29) | 37 (45) | 5 (8) | <0.05 |
| **Neurological complications** | | | | |
| Seizures, n (%) | 52 (36) | 36 (44) | 16 (26) | 0.04 |
| Rebleeding, n (%) | 14 (10) | 4 (5) | 10 (16) | 0.04 |
| Hydrocephalus, n (%) | 95 (66) | 67 (82) | 28 (46) | 0.001 |
| DCI, n (%) | 56 (39) | 28 (34) | 28 (46) | 0.17 |
| Intracranial hypertension, n (%) | 85 (59) | 46 (57) | 39 (64) | 0.39 |
| **Outcomes** | | | | |
| ICU LOS- days (IQR) | 16 (9-22) | 15 (9-21) | 17 (9-26) | 0.19 |
| Hospital LOS- days (IQR) | 26 (13-52) | 27 (13- 49) | 24 (10-55) | 0.83 |
| GOS -points, median (IQR) | 3 (1-4) | 3 (1-5) | 2 (1-4) | 0.24 |
| Unfavorable outcome, n (%) | 87 (61) | 50 (61) | 37 (61) | 0.99 |
| ICU death, n (%) | 59 (41) | 29 (35) | 30 (49) | 0.12 |
| Hospital death, n (%) | 62 (43) | 32 (39) | 30 (49) | 0.24 |

N=number; IQR: interquartile range; APACHE : acute physiology and chronic health evaluation; SOFA : sequential organ failure assessment; GCS: Glasgow coma scale; WFNS: world federation of neurological surgeons; COPD: chronic obstructive pulmonary disease; RRT: renal replacement therapy; ECMO: extra-corporeal membrane oxygenation. PbtO_2_: brain tissue oxygenation; DCI: delayed cerebral ischemia; ICU: intensive care unit; LOS: length of stay; GOS: Glasgow outcome scale.

**Supplemental Table S8:** Characteristics of the study population, including only aneurysmal SAH and according to mortality and neurological outcome. Data are presented as count (%), mean ±SD or median (IQRs).

|  | **Survivors**  **N=81** | **Non-survivors**  **N=62** | **p value** | **FO**  **(N=56)** | **UO**  **(N=87)** | **p value** |
| --- | --- | --- | --- | --- | --- | --- |
| Age, years | 54 (±11) | 56 (±15) | 0.36 | 52 (±11) | 53 (±14) | 0.06 |
| Male gender, n (%) | 34 (42) | 20 (32) | 0.30 | 27 (48) | 27 (31) | 0.05 |
| APACHE on admission | 17 (12-21) | 20 (15-22) | 0.02 | 16 (11-19) | 20 (15-23) | 0.001 |
| SOFA on admission | 6 (4-9) | 9 (5-10) | 0.03 | 6 (3-8) | 9 (5-10) | 0.002 |
| GCS on admission | 7 (3-13) | 3 (3-10) | 0.13 | 7 (3-13) | 4 (3-9) | 0.02 |
| Poor grade (WFNS 4-5), n (%) | 60 (74) | 50 (81) | 0.43 | 38 (68) | 72 (83) | 0.04 |
| High risk modified Fisher scale, n (%) | 78 (96) | 57 (92) | 0.29 | 54 (96) | 81 (93) | 0.48 |
| Anterior location aneurysm, n (%) | 60 (74) | 45 (73) | 0.85 | 39 (70) | 66 (76) | 0.44 |
| Intraparenchymal hematoma, n (%) | 28 (35) | 39 (47) | 0.17 | 15 (27) | 42 (48) | 0.01 |
| Hypertension, n (%) | 44 (54) | 26 (42) | 0.18 | 28 (50) | 42 (48) | 0.87 |
| Diabetes mellitus, n (%) | 9 (11) | 5 (8) | 0.59 | 7 (13) | 7 (8) | 0.40 |
| Heart disease, n (%) | 9 (11) | 6 (10) | 0.99 | 4 (7) | 11 (13) | 0.41 |
| Previous neurological disease, n (%) | 7 (9) | 7 (11) | 0.78 | 6 (11) | 8 (9) | 0.78 |
| Chronic Kidney Disease, n (%) | 3 (4) | 0 | 0.26 | 2 (4) | 1 (1) | 0.56 |
| COPD, n (%) | 4 (5) | 11 (18) | 0.03 | 4 (7) | 11 (13) | 0.41 |
| Immunosuppression, n (%) | 3 (4) | 3 (5) | 0.99 | 1 (2) | 5 (6) | 0.40 |
| Cancer, n (%) | 1 (1) | 6 (10) | 0.04 | 1 (2) | 6 (7) | 0.25 |
| Cirrhosis, n (%) | 2 (3) | 3 (5) | 0.65 | 1 (2) | 4 (5) | 0.65 |
| Vasopressors, n (%) | 53 (65) | 56 (90) | 0.001 | 34 (61) | 75 (86) | 0.001 |
| Inotropic agents, n (%) | 21 (26) | 24 (39) | 0.15 | 14 (25) | 31 (36) | 0.20 |
| Mechanical ventilation, n (%) | 67 (83) | 62 (100) | 0.001 | 46 (82) | 83 (95) | 0.02 |
| RRT, n (%) | 0 | 0 | - | 0 | 0 | - |
| ECMO, n (%) | 0 | 2 (3) | 0.19 | 0 | 2 (2) | 0.52 |
| Endovascular treatment (coiling), n (%) | 65 (80) | 45 (73) | 0.32 | 47 (84) | 63 (72) | 0.15 |
| Surgical treatment (clipping), n (%) | 14 (17) | 19 (31) | 0.07 | 5 (9) | 28 (32) | 0.001 |
| Nimodipine (prophylaxis), n (%) | 73 (90) | 52 (84) | 0.31 | 54 (96) | 78 (82) | 0.009 |
| ICP/PbtO_2_ guided therapy | 31 (38) | 30 (48) | 0.24 | 24 (43) | 37 (43) | 0.99 |
| Seizures, n (%) | 30 (37) | 22 (36) | 0.86 | 16 (29) | 36 (41) | 0.15 |
| Re-bleeding, n (%) | 3 (4) | 11 (18) | 0.009 | 1 (2) | 13 (15) | 0.009 |
| Hydrocephalus, n (%) | 59 (73) | 36 (58) | 0.08 | 41 (73) | 54 (62) | 0.21 |
| DCI, n (%) | 25 (31) | 31 (50) | 0.03 | 14 (25) | 42 (48) | 0.008 |
| Intracranial hypertension, n (%) | 31 (38) | 55 (89) | 0.001 | 16 (29) | 70 (80) | 0.001 |
| Induced Hypertension, n (%) | 51 (63) | 37 (60) | 0.73 | 34 (61) | 54 (62) | 0.99 |
| Osmotic therapy, n (%) | 24 (30) | 44 (71) | 0.001 | 10 (18) | 58 (67) | 0.001 |
| Barbituric coma, n (%) | 9 (11) | 25 (40) | 0.001 | 4 (7) | 30 (35) | 0.001 |
| Hypothermia, n (%) | 8 (10) | 22 (36) | 0.001 | 4 (7) | 26 (30) | 0.001 |
| Decompressive craniectomy, n (%) | 7 (9) | 6 (10) | 0.99 | 3 (5) | 10 (11) | 0.25 |
| Intra-arterial nimodipine, n (%) | 30 (37) | 22 (36) | 0.86 | 21 (38) | 31 (36) | 0.86 |
| Cerebral angioplasty, n (%) | 14 (17) | 7 (11) | 0.35 | 9 (16) | 12 (14) | 0.81 |
| ICU LOS, days | 19 (15-26) | 10 (5-15) | 0.001 | 18 (14-23) | 13 (6-21) | 0.001 |
| Hospital LOS, days | 46 (29-84) | 11 (5-16) | 0.001 | 39 (27-75) | 15 (7-33) | 0.001 |

APACHE : acute physiology and chronic health evaluation; SOFA : sequential organ failure assessment; GCS: Glasgow coma scale WFNS: world federation of neurological surgeons; COPD: chronic obstructive pulmonary disease; RRT: renal replacement therapy; ECMO: extra-corporeal membrane oxygenation. PbtO_2_: brain tissue oxygenation; DCI: delayed cerebral ischemia; ICHT: Intracranial hypertension; ICU: intensive care unit; LOS: length of stay.

**Supplemental** **Table S9:** Logistic regression analysis to identify variables independently associated with 6-month unfavorable neurologic outcome, including only aneurysmal SAH. Data are reported as odds ratio (OR) and 95 % confidence intervals (CIs).

|  | **Univariable analysis**  **OR (95% CI)** | **P value** | **Multivariable analysis**  **OR (95% CI)** | **P value** |
| --- | --- | --- | --- | --- |
| **Age** | 1.03 (1.00-1.06) | 0.07 | 1.06 (1.02-1.10) | 0.004 |
| **Poor Grade (WFNS 4-5)** | 2.27 (1.03-5.01) | 0.04 | 1.71 (0.62-4.68) | 0.30 |
| **Intracranial hypertension** | 9.58 (4.40-20.86) | 0.001 | 10.51 (4.15-26.59) | 0.001 |
| **DCI** | 2.80 (1.34-5.85) | 0.006 | 6.23 (2.20-17.70) | 0.001 |
| **Endovascular treatment** | 0.50 (0.21-1.18) | 0.11 | 0.59 (0.18-2.02) | 0.40 |
| **Nimodipine prophylaxis** | 0.16 (0.04-0.75) | 0.02 | 0.11 (0.02-0.72) | 0.02 |
| **Intraparenchymal Hematoma** | 2.55 (1.24-5.27) | 0.01 | 2.66 (0.98-7.19) | 0.06 |
| **ICP/PbtO_2_ guided therapy** | 0.99 (0.50-1.95) | 0.97 | 0.44 (0.17-1.16) | 0.10 |

WFNS : world federation of neurological surgeons; ICP: intracranial hypertension; PbtO_2_: brain tissue oxygenation; DCI = delayed cerebral ischemia.

**Supplemental Table 10:** Cox regression to identify variables independently associated with hospital mortality, including only aneurysmal SAH. Data are reported as hazard ratio (HR) and 95 % confidence intervals (CIs).

|  | **Univariable analysis**  **HR (95% CI)** | **P value** | **Multivariable analysis**  **HR (95% CI)** | **P value** |
| --- | --- | --- | --- | --- |
| **APACHE** | 1.06 (1.01-1.12) | 0.01 | 1.06 (1.01-1.11) | 0.02 |
| **Nimodipine** | 0.57 (0.29-1.12) | 0.10 | 0.83 (0.41-1.69) | 0.61 |
| **Endovascular treatment** | 0.64 (0.37-1.12) | 0.12 | 0.52 (0.28-0.96) | 0.04 |
| **Intracranial hypertension** | 6.81 (3.23-14.35) | 0.001 | 6.77 (3.14-14.59) | 0.001 |
| **DCI** | 1.50 (0.91-2.47) | 0.11 | 1.18 (0.71-1.97) | 0.53 |
| **Intraparenchymal hematoma** | 1.45 (0.88-2.40) | 0.14 | 0.87 (0.55-1.64) | 0.61 |
| **ICP/PbtO_2_ guided therapy** | 1.32 (0.80-2.17) | 0.28 | 1.35 (0.82-2.24) | 0.24 |

ICP: intracranial pressure; PbtO_2_: brain tissue oxygenation; APACHE: acute physiology and chronic health evaluation; DCI: delayed cerebral ischemia

**Supplemental Table S11:** Characteristics of the patients undergoing ICP-guided or ICP/PbtO_2_ guided therapy, including only aneurysmal SAH. Data are presented as count (%), mean ±SD or median (IQRs).

|  | **All treated patients (N=95)** | **ICP**  **(N=42)** | **ICP/PbtO_2_**  **(N=53)** | **p value** |
| --- | --- | --- | --- | --- |
| **On Admission** | | | | |
| Age, mean (±SD) | 54 (±13) | 55 (±13) | 54 (±13) | 0.80 |
| Male gender, n (%) | 35 (37) | 14 (33) | 21 (53) | 0.70 |
| APACHE score, median (IQR) | 19 (14-22) | 19 (15-22) | 18 (13-21) | 0.16 |
| SOFA score, median (IQR) | 8 (5-10) | 8 (5-10) | 8 (3-10) | 0.24 |
| GCS, median (IQR) | 5 (3-7) | 6 (3-13) | 5 (3-11) | 0.35 |
| WFNS 4-5, n (%) | 75 (79) | 37 (88) | 38 (72) | 0.08 |
| mFisher scale 3 or 4 points, n (%) | 88 (93) | 41 (98) | 47 (89) | 0.13 |
| Intraparenchymal hematoma, n (%) | 46 (48) | 21 (50) | 25 (47) | 0.84 |
| Anterior circulation aneurysm, n (%) | 63 (66) | 39 (93) | 24 (45) | 0.001 |
| **Comorbidities** | | | | |
| HAS, n (%) | 42 (44) | 22 (52) | 20 (39) | 0.21 |
| DM, n (%) | 9 (10) | 5 (12) | 4 (8) | 0.50 |
| Heart disease, n (%) | 10 (11) | 6 (14) | 4 (8) | 0.33 |
| Previous neuro disease, n (%) | 14 (10) | 6 (14) | 3 (6) | 0.18 |
| CKD, n (%) | 0 | 0 | 0 | - |
| Asthma /COPD, n (%) | 9 (10) | 3 (7) | 6 (11) | 0.73 |
| Immunosuppression, n (%) | 4 (4) | 3 (7) | 1 (2) | 0.32 |
| Cancer, n (%) | 4 (4) | 2 (5) | 2 (4) | 0.99 |
| Cirrhosis, n (%) | 3 (3) | 2 (5) | 1 (2) | 0.58 |
| **Support therapies during ICU stay** | | | | |
| Vasopressor, n (%) | 85 (90) | 34 (81) | 51 (96) | 0.02 |
| Inotropic, n (%) | 40 (42) | 4 (10) | 36 (68) | 0.001 |
| Mechanical ventilation, n(%) | 91 (96) | 40 (95) | 51 (96) | 0.99 |
| RRT, n (%) | 0 | 0 | 0 | - |
| ECMO, n (%) | 1 (1) | 0 | 1 (2) | 0.99 |
| **Treatments** | | | | |
| Surgical clipping, n (%) | 26 (27) | 13 (31) | 13 (25) | 0.50 |
| Endovascular coiling, n (%) | 70 (74) | 29 (69) | 41 (77) | 0.48 |
| Nimodipine (prophylaxis), n (%) | 80 (84) | 34 (81) | 46 (87) | 0.58 |
| Osmotic therapy , n (%) | 68 (72) | 42 (100) | 26 (49) | 0.001 |
| Induced Hypertension, n (%) | 64 (67) | 21 (50) | 43 (81) | 0.002 |
| Barbituric coma, n (%) | 17 (18) | 17 (41) | 17 (32) | 0.52 |
| Induced hypothermia, n (%) | 29 (31) | 11 (26) | 18 (34) | 0.50 |
| Decompressive craniectomy, n (%) | 13 (14) | 5 (12) | 8 (15) | 0.77 |
| Intra-arterial nimodipine, n (%) | 42 (44) | 10 (24) | 32 (60) | 0.001 |
| Angioplasty, n (%) | 17 (18) | 6 (14) | 11 (20) | 0.59 |
| **Neurological complications** | | | | |
| Seizures, n (%) | 33 (35) | 18 (43) | 15 (28) | 0.19 |
| Rebleeding, n (%) | 12 (13) | 4 (10) | 8 (15) | 0.54 |
| Hydrocephalus, n (%) | 54 (57) | 30 (71) | 24 (45) | 0.02 |
| DCI, n (%) | 45 (47) | 17 (41) | 28 (53) | 0.30 |
| Intracranial hypertension, n (%) | 76 (79) | 42 (100) | 34 (64) | 0.001 |
| **Outcomes** | | | | |
| ICU LOS- days (IQR) | 16 (9-22) | 15 (9-21) | 17 (9-26) | 0.19 |
| Hospital LOS- days (IQR) | 26 (13-52) | 27 (13- 49) | 24 (10-55) | 0.83 |
| GOS -points, median (IQR) | 1 (1-4) | 1 (1-3) | 2 (1- 4) | 0.08 |
| Unfavorable outcome, n (%)* | 69 (73) | 36 (86) | 33 (62) | 0.01 |
| ICU death, n (%) | 52 (55) | 26 (62) | 26 (49) | 0.22 |
| Hospital death, n (%) | 52 (55) | 26 (39) | 26 (49) | 0.22 |

**Supplemental Table S12 :** Characteristics of the patients undergoing ICP- or ICP/PbtO_2_ guided-therapy, including only aneurysmal SAH and according to hospital mortality and neurological outcome. Data are presented as count (%), mean ±SD or median (IQRs).

|  | **Survivors**  **N=43** | **Non-survivors**  **N=52** | **p value** | **FO**  **N=26** | **UO**  **N=69** | **p value** |
| --- | --- | --- | --- | --- | --- | --- |
| Age, years, mean (±SD) | 54 (±11) | 54 (±14) | 0.98 | 52 (±11) | 55 (±13) | 0.28 |
| Male gender, n (%) | 17 (40) | 18 (35) | 0.67 | 12 (46) | 23 (33) | 0.34 |
| APACHE on admission, median (IQR) | 18 (13-21) | 20 (15-22) | 0.13 | 17 (11-20) | 19 (15-22) | 0.05 |
| SOFA on admission, median (IQR) | 8 (5-10) | 9 (4-10) | 0.77 | 7 (4-10) | 9 (5-10) | 0.34 |
| GCS on admission, median (IQR) | 6 (3-13) | 3 (3-10) | 0.27 | 7 (3-13) | 5 (3-8) | 0.21 |
| Poor grade (WFNS 4-5), n (%) | 32 (74) | 43 (52) | 0.45 | 17 (65) | 58 (84) | 0.09 |
| High risk modified Fisher scale, n (%) | 41 (95) | 47 (90) | 0.45 | 25 (96) | 63 (91) | 0.67 |
| Anterior location aneurysm, n (%) | 28 (67) | 35 (67) | 0.83 | 17 (65) | 49 (71) | 0.15 |
| Intraparenchymal hematoma, n (%) | 19 (44) | 27 (52) | 0.54 | 9 (35) | 37 (54) | 0.11 |
| Hypertension, n (%) | 23 (54) | 19 (37) | 0.15 | 12 (46) | 30 (44) | 0.82 |
| Diabetes mellitus, n (%) | 6 (14) | 3 (6) | 0.29 | 4 (15) | 5 (7) | 0.25 |
| Heart disease, n (%) | 5 (12) | 5 (10) | 0.75 | 2 (8) | 8 (12) | 0.72 |
| Previous neurological disease, n (%) | 3 (7) | 6 (12) | 0.51 | 3 (12) | 6 (9) | 0.70 |
| Chronic Kidney Disease, n (%) | 0 | 0 | - | 0 | 0 | - |
| COPD, n (%) | 2 (5) | 7 (14) | 0.18 | 2 (8) | 7 (10) | 0.99 |
| Immunosuppression, n (%) | 1 (2) | 3 (6) | 0.62 | 0 | 4 (6) | 0.57 |
| Cancer, n (%) | 0 | 4 (8) | 0.62 | 0 | 4 (6) | 0.57 |
| Cirrhosis, n (%) | 1 (2) | 2 (4) | 0.99 | 0 | 3 (4) | 0.56 |
| Vasopressors, n (%) | 36 (84) | 49 (94) | 0.18 | 23 (89) | 62 (90) | 0.99 |
| Inotropic agents, n (%) | 19 (44) | 21 (40) | 0.84 | 13 (50) | 27 (39) | 0.36 |
| Mechanical ventilation, n (%) | 39 (91) | 52 (100) | 0.04 | 24 (92) | 67 (97) | 0.30 |
| RRT, n (%) | 0 | 0 | - | 0 | 0 | - |
| ECMO, n (%) | 0 | 1 (2) | 0.99 | 0 | 1 (1) | 0.99 |
| Endovascular treatment (coiling), n (%) | 34 (79) | 36 (69) | 0.35 | 23 (89) | 47 (68) | 0.07 |
| Surgical treatment (clipping), n (%) | 8 (19) | 18 (35) | 0.11 | 1 (4) | 25 (36) | 0.001 |
| Nimodipine (prophylaxis), n (%) | 37 (86) | 43 (83) | 0.78 | 25 (96) | 55 (80) | 0.06 |
| PbtO_2_ guided therapy, n (%) | 27 (63) | 26 (50) | 0.22 | 20 (77) | 33 (48) | 0.01 |
| Seizures, n (%) | 16 (37) | 17 (33) | 0.67 | 8 (31) | 25 (36) | 0.81 |
| Re-bleeding, n (%) | 3 (7) | 9 (17) | 0.21 | 1 (4) | 11 (16) | 0.17 |
| Hydrocephalus, n (%) | 25 (58) | 29 (56) | 0.84 | 15 (54) | 40 (58) | 0.82 |
| DCI, n (%) | 19 (44) | 26 (50) | 0.68 | 10 (39) | 35 (51) | 0.36 |
| Intracranial hypertension, n (%) | 26 (61) | 50 (96) | 0.001 | 13 (50) | 63 (91) | 0.001 |
| Induced Hypertension, n (%) | 33 (77) | 31 (60) | 0.08 | 20 (77) | 44 (64) | 0.133 |
| Osmotic therapy, n (%) | 24 (56) | 44 (85) | 0.003 | 10 (39) | 58 (84) | 0.001 |
| Barbituric coma, n (%) | 9 (21) | 25 (48) | 0.009 | 4 (15) | 30 (44) | 0.02 |
| Hypothermia, n (%) | 24 (56) | 21 (40) | 0.63 | 4 (15) | 25 (36) | 0.08 |
| Decompressive craniectomy, n (%) | 7 (16) | 6 (12) | 0.56 | 3 (12) | 10 (15) | 0.99 |
| Intra-arterial nimodipine, n (%) | 24 (56) | 18 (35) | 0.06 | 17 (65) | 25 (36) | 0.02 |
| Cerebral angioplasty, n (%) | 13 (30) | 4 (8) | 0.006 | 8 (31) | 9 (13) | 0.07 |
| ICU LOS, days | 25 (19-33) | 10 (5-16) | 0.001 | 25 (17-30) | 13 (6-20) | 0.001 |
| Hospital LOS, days | 52 (34-89) | 10 (5-16) | 0.001 | 49 (33-83) | 14 (6-28) | 0.001 |

APACHE : acute physiology and chronic health evaluation; SOFA : sequential organ failure assessment; GCS: Glasgow coma scale WFNS: world federation of neurological surgeons; COPD: chronic obstructive pulmonary disease; RRT: renal replacement therapy; ECMO: extra-corporeal membrane oxygenation. PbtO_2_: brain tissue oxygenation; DCI: delayed cerebral ischemia; ICHT: Intracranial hypertension; ICU: intensive care unit; LOS: length of stay.

**Supplemental Table S13**: Logistic regression analysis to identify possible association between combined ICP/PbtO_2_ guided therapy and 6-month unfavorable neurologic outcome in patients undergoing ICP- or ICP/PbtO_2_ guided-therapy (n=95). This analysis included only aneurysmal SAH. Data are reported as odds ratio (OR) and 95 % confidence intervals (CIs).

| **Model 1** | **Univariable analysis**  **OR (95% CI)** | **P value** | **Multivariable analysis**  **OR (95% CI)** | **P value** |
| --- | --- | --- | --- | --- |
| **ICP/PbtO_2_ guided therapy** | 0.28 (0.10-0.77) | 0.02 | 0.29 (0.10-0.83) | 0.02 |
| **Nimodipine prophylaxis** | 0.16 (0.02-1.26) | 0.08 | 0.22 (0.03-1.94) | 0.14 |
| **Endovascular therapy** | 0.28 (0.08-1.03) | 0.06 | 0.39 (0.10-1.52) | 0.18 |
| **Model 2** | **Univariable analysis**  **OR (95% CI)** | **P value** | **Multivariable analysis**  **OR (95% CI)** | **P value** |
| **ICP/PbtO_2_ guided therapy** | 0.28 (0.10-0.77) | 0.02 | 0.31 (0.11-0.91) | 0.03 |
| **Nimodipine prophylaxis** | 0.16 (0.02-1.26) | 0.08 | 0.25 (0.03-2.20) | 0.18 |
| **Endovascular therapy** | 0.28 (0.08-1.03) | 0.06 | 0.40 (0.10-1.61) | 0.20 |
| **WFNS 4-5** | 2.79 (0.99-7.85) | 0.05 | 1.90 (0.64-5.69) | 0.29 |
| **Model 3** | **Univariable analysis**  **OR (95% CI)** | **P value** | **Multivariable analysis**  **OR (95% CI)** | **P value** |
| **ICP/PbtO_2_ guided therapy** | 0.28 (0.10-0.77) | 0.02 | 0.32 (0.11-0.92) | 0.03 |
| **Nimodipine prophylaxis** | 0.16 (0.02-1.26) | 0.08 | 0.25 (0.03-2.19) | 0.19 |
| **Endovascular therapy** | 0.28 (0.08-1.03) | 0.06 | 0.38 (0.10-1.54) | 0.18 |
| **APACHE score** | 1.08 (1.00-1.17) | 0.06 | 1.06 (0.97-1.15) | 0.18 |

APACHE : acute physiology and chronic health evaluation; WFNS: world federation of neurological surgeons; PbtO_2_: brain tissue oxygenation; ICP : intracranial pressure

**Supplemental Table S14:** Cox regression to identify variables independently associated with hospital mortality in patients undergoing ICP- or PbtO_2_ guided-therapy (n=95), including only aneurysmal SAH. Data are presented as hazard ratio (HR) and 95% confidence intervals (CI).

|  | **Univariable analysis**  **HR ( 95% CI)** | **P value** | **Multivariable analysis**  **HR (95% CI)** | **P value** |
| --- | --- | --- | --- | --- |
| **APACHE score** | 1.06 (1.01-1.12) | 0.03 | 1.06 (1.01-1.12) | 0.03 |
| **Combined ICP/PbtO_2_ guided therapy** | 0.82 (0.47-1.42) | 0.47 | 0.86 (0.49-1.50) | 0.59 |
| **Surgical treatment (clipping)** | 1.96 (1.10-3.47) | 0.02 | 1.58 (0.86-2.90) | 0.14 |
| **Re-bleeding** | 2.32 (1.13-4.79) | 0.02 | 2.27 (1.05-4.89) | 0.04 |

ICP: intracranial hypertension; PbtO_2_: brain tissue oxygenation; APACHE: acute physiology and chronic health evaluation
